# Evaluation of Strength Properties of Sand Modified with Organic Polymers

**DOI:** 10.3390/polym10030287

**Published:** 2018-03-08

**Authors:** Jin Liu, Yuxia Bai, Zezhuo Song, Yi Lu, Wei Qian, Debi Prasanna Kanungo

**Affiliations:** 1 School of Earth Sciences and Engineering, Hohai University, Nanjing 210098, China; byxhhu@163.com (Y.B.); szzhhu@163.com (Z.S.); wei.geoserve@gmail.com (W.Q.); 2 Key Laboratory of Earth Fissures Geological Disaster, Ministry of Land and Resource (Geological Survey of Jiangsu Province), Nanjing 210049, China; lynju@163.com; 3 Council of Scientific and Industrial Research (CSIR)—Central Building Research Institute (CBRI), Roorkee 247667, India; debi.kanungo@gmail.com

**Keywords:** organic polymers, sand, strength behaviour, laboratory test

## Abstract

Due to weak physical properties of sand, chemical reinforcement methods are widely used to improve sand properties to meet the engineering requirements. However, most of the traditional additives cause environmental problems. Therefore, non-traditional additives such as liquid polymers, enzymes, ions, and lignin derivatives have been studied extensively. In this study, organic polymer is used as a soil stabilizer to reinforce the sand. To evaluate the effectiveness of the organic polymer as soil stabilizer, a series of unconfined compression strength (UCS) tests, direct shear tests, and tensile tests were carried out on reinforced sand with different polymer concentrations and dry densities of sand. The reinforcement mechanism was analysed with scanning electron microscopy (SEM) images. The results indicated that the polymer concentration and dry density of sand had significant effects on the strength characteristics of reinforced sand specimens. The unconfined compressive strength, cohesion, and tensile strength of specimens with the same dry density increased with the increasing polymer concentration. The polymer membranes—formed by the mixture of polymer and water—enwrap the sand particles and interlink them to form a stable structure. The efficiency of this stabilization changed with dry sand density.

## 1. Introduction

Soil reinforcement has been widely used in geotechnical engineering. Chemical reinforcement for soil improvement is a popular method using additives such as traditional stabilizers and non-traditional additives. The traditional chemical stabilizers such as lime, fly ash, zeolite gypsum, cement, and other industrial wastes have more advantages for the improvement of soil strength [1,2,3,4], but most of the traditional additives easily increase the pH value of soil, resulting in the environmental problems of polluting ground water or obstructing vegetation growth [5,6]. Recently, non-traditional chemical additives such as liquid polymers, enzymes, ions, and lignin derivatives have been studied [7,8,9]. Polymeric material as a non-traditional soil stabilizer has been developed to improve the physical and engineering properties of soil to meet the requirements of different engineering practices. 

Sands are usually problematic in many types of geotechnical engineering applications in foundation and slope stabilization because of their relatively low strength, loose structure, and/or powerful saturated liquefaction potential. The polymer materials used for sand stabilization have been studied by many researchers. Many types of polymers as reinforcement materials have been studied systematically by laboratory test [10,11,12,13,14,15]. Naeini et al. [16] studied the effect of epoxy resin on the compressive strength and modulus of elasticity of samples under wet and dry conditions. Martoet et al. [17] discussed the influences of TX-85 and SH-85 additives on the engineering and micro-structural characteristics of lateritic soil. Sun et al. [18] introduced xanthan gum—a type of environmentally-friendly organic polymer—to promote the growth of vegetation in coastal sand dunes, thereby reducing the price of coastal erosion protection. Yang et al. [19] studied the effects of aging tests on a novel chemical sand-fixing agent (polyaspartic acid resin). These polymer materials can change the loose structure and improve the strength by filling up the void spaces and enwrapping the sand particles [20,21,22]. These research results indicated that the polymeric materials can be considered as a soil stabilizer to improve the strength characteristics of sand.

The goal of this study is to present the results from a series of unconfined compression strength (UCS) tests, direct shear tests, and tensile tests conducted on untreated and organic polymer-reinforced sand to examine the improvement in the strength characteristics of reinforced sand. Different polymer concentrations and sand dry densities were chosen to study the effect of polymer on sand improvement. SEM was used to study the micro-characterization of sand–polymer. The results and associated discussion provide the chemical stabilization mechanisms of polymer-reinforced sand for researchers and practicing engineers.

## 2. Experimental Outlines

### 2.1. Materials

#### 2.1.1. Sand

**Figure 1:**
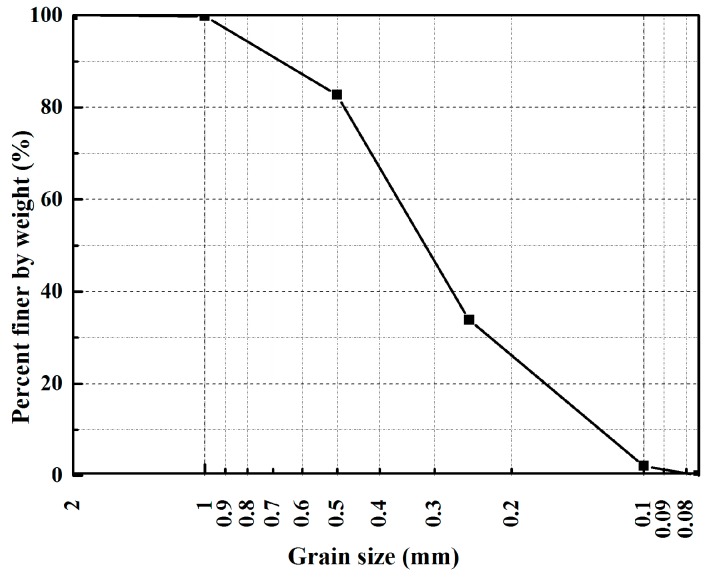
Grain size distribution of sand.

The sand used in the tests was obtained from Nanjing city of Jiangsu province, China. Its particle size distribution is shown in Figure 1. It has a specific gravity (*G*s) of 2.64, maximum dry density (ρ_max_) of 1.66 g/cm^3^, minimum dry density (ρ_min_) of 1.34 g/cm^3^, maximum void ratio (*e*_max_) of 0.97, minimum void ratio (*e*_min_) of 0.59. Its particles have a mean grain size (*D*_50_) of 0.30 mm, gradation coefficient (*C*_g_) of 1.13, and uniformity coefficient (*C*_u_) of 2.77.

#### 2.1.2. Organic Polymers

**Figure 2:**
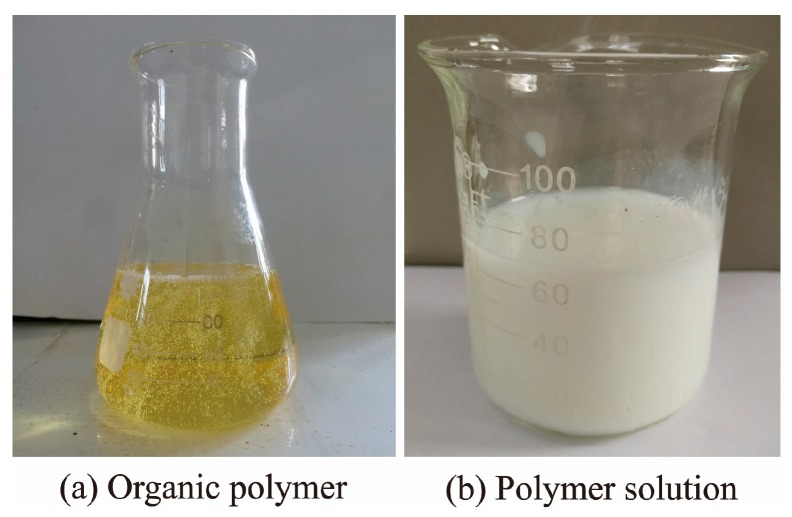
Photo of (**a**) Organic polymer; and (**b**) Polymer solution.

The organic polymer used as soil stabilizer in this study has a main constituent of polyurethane resin. The polymerization process of this organic polymer is as follows. Firstly, the weight ratio (5:5:2:17) of poly-oxypropylene diol (PPG, Jining Hongming Chemical Reagent Co., Ltd., Jining, China) with molecular weight 2000, poly-oxyethylene glycol (PEG, Shanghai Ika Biotechnology Co., Ltd., Shanghai, China) with molecular weight 2000, poly-caprolaclone glycol (PCL, Jining Hongming Chemical Reagent Co., Ltd., Jining, China) with molecular weight 1000, and toluene were mixed with the conditions of temperature 145 °C and air distillation. After most of the toluene was dried, the vacuum distillation was undertaken to remove the residual water and toluene completely. Secondly, the temperature was cooled to 20 °C, it was placed in a reflux condenser, N_2_ was added, and the reaction system was sealed with oil. Toluene diisocynate (TDI, Nantong Runfeng Petrochemical Co., Ltd., Nantong, China) with two times weight of diols was added and the reaction was undertaken with temperature 90 °C for 2 h. Finally, the reaction temperature was reduced to 20 °C and ethyl acetate (Nantong Taichang Chemical Materials Co., Ltd., Suzhou, China) was added with stirring for 1 h. After that, lauryl sodium sulphate (Qidong Mingcheng Chemical Co., Ltd., Nantong, China) was mixed according to the definite weight percentage of polyurethane prepolymer to obtain the final organic polymer, a light-yellow liquid with a pH of about 7, specific gravity of 1.15 g/cm^3^, viscosity of 650–800 mPa·s, solid content of 88%, coagulation time of 60–1600 s. The organic polymer solution in this study was prepared with distilled water. The mixture of liquid polymer (Figure 2a) and distilled water were stirred for about 10 min to obtain the desired solution (Figure 2b).

### 2.2. Experimental Methods

The strength properties are important factors to evaluate the effectiveness of sand reinforcement. In this study, laboratory trials of unconfined compression test, direct shear test, and pull-out test were performed to evaluate the performance of polymer-reinforced sand. In the three types of tests, sand samples were first oven-dried. Five concentrations (10%, 20%, 30%, 40%, and 50%) of polymer dilutions were proposed for sand reinforcement, with water (0%) as a control. The three dry densities of the prepared samples were 1.40, 1.50, and 1.60 g/cm^3^, respectively. The additive amount of polymer dilutions was 10% by weight of dry sand. 

#### 2.2.1. Unconfined Compression Test

**Figure 3:**
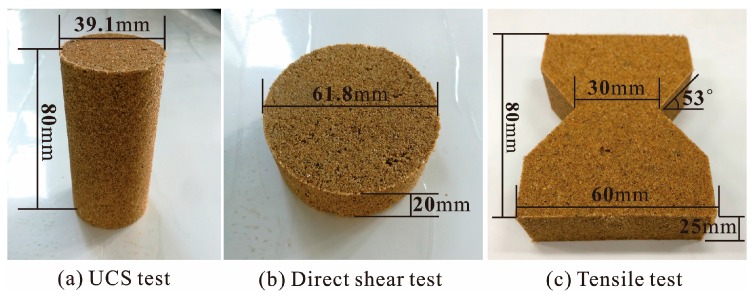
Photo of three tests reinforced specimens: (**a**) Unconfined compression strength (UCS) test; (**b**) Direct shear test; (**c**) Tensile test.

**Figure 4:**
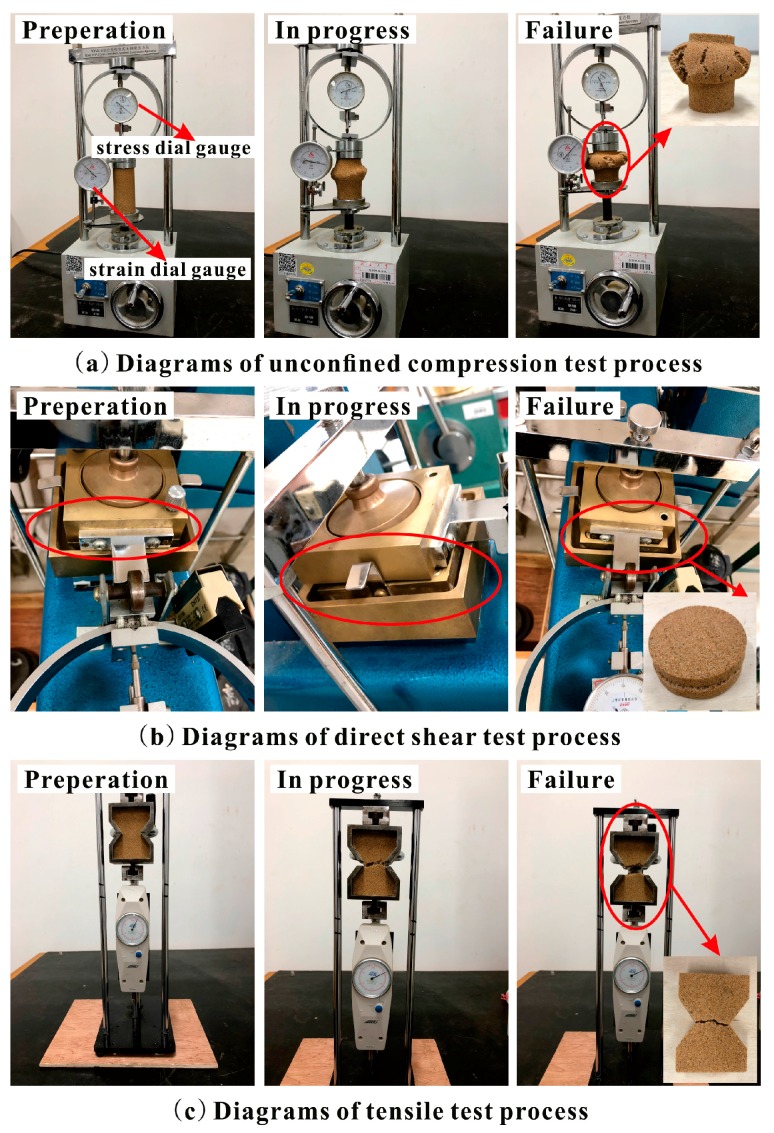
Diagrams of three tests: (**a**) UCS test; (**b**) Direct shear test; (**c**) Tensile test.

In the unconfined compression test, the dried sand was mixed with the proposed polymer dilution and then prepared with the static compaction method based on ASTM standards (ASTM D2166-00) [23]. Four-layered compaction was adopted to keep the uniformity of test specimens (Figure 3a) with the diameter being 39.1 mm and height being 80 mm. After the specimen preparation, the reinforced specimens were kept in a curing box with a temperature of around 20 °C and curing time of 48 h. The unconfined compression tests were carried out with a YYW-2 unconfined compressional strain control device (Figure 4a) at a loading rate of 2.4 mm/min until sample failure. Additionally, the unconfined compression test was performed on specimen triplicates and average values were used. The axial stress and strain were recorded by stress dial gauge (Figure 4a) and strain dial gauge (Figure 4a), respectively. The peak strength, residual strength, axial strain of failure (ε_f_), elasticity modulus (*E*), and failure modulus (*E*_f_) were obtained. In this study, the specimen was observed to be failed when the axial stress reached the peak value. Failure modulus is the ratio of peak strength to its corresponding axial strain (ε_f_). Elasticity modulus is the ratio of axial stress (which is half of peak strength), to its corresponding axial strain.

#### 2.2.2. Direct Shear Test

In the direst shear test, the dried sand was mixed with the proposed polymer dilution and then prepared with the static compaction method based on ASTM standards (ASTM D3080-98) [24]. Two-layered compaction was adopted to test specimens (Figure 3b) with 61.8 mm diameter and 20 mm height. After the specimen preparation, the reinforced specimens were kept in a curing box with a temperature of around 20 °C and curing time 48 h. The direct shear tests were carried out with a ZJ typed strain control direct shear apparatus (Figure 4b) at a strain rate of 0.8 mm/min under the normal pressures of 50, 100, 200, and 300 kPa to obtain the maximum shear stress (τ). Based on Coulomb’s law of shear strength (τ = σ ∙ tanø + c), shear stress parameters (c and ø) were calculated. Four shear strength values and four normal pressures were used to fit a straight line. Intercept and slope are c and tanø. The shear strength parameters were obtained on specimen triplicates, and average values were used.

#### 2.2.3. Tensile Test

Tensile tests were carried out with a self-manufactured testing device (Figure 4c). The specimen of the pull-out test is shown in (Figure 3c) and was prepared with the static compaction method. After the specimen preparation, the reinforced specimens were kept in a curing box with a temperature of around 20 °C and curing time of 48 h. The tensile force of the middle section of specimen was measured to calculate the tensile strength. The specimen triplicates were taken, and their average values were used.

## 3. Test Results

The specimens with five polymer concentrations and three densities were tested in unconfined compression test, direct shear test and tensile test. Diagrams of the tests process are shown in Figure 4. Their results are summarized in Table 1, Table 2 and Table 3. The detailed analysis of each test is given as follows.

### 3.1. Unconfined Compression Test

**Figure 5:**
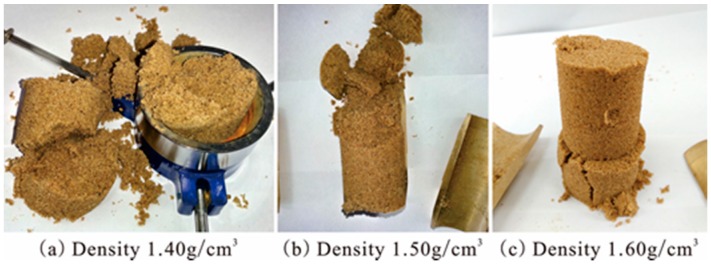
The shape of unreinforced specimens in unconfined compression test: (**a**) Density 1.40 g/cm^3^; (**b**) Density 1.50 g/cm^3^; (**c**) Density 1.60 g/cm^3^.

All the testing conditions and their results in unconfined compression tests are listed in Table 1. It was very difficult to form the required specimens of the unreinforced sand with smaller cohesive force for testing in unconfined conditions. The unreinforced specimens with water content of 10% were prepared in moulds with static compaction method to the designed densities of 1.40, 1.50, and 1.60 g/cm^3^ respectively. After stripping the specimen mould, the shapes of specimens are presented in Figure 5. As seen, all the specimens generated disintegration in different degrees. The specimen with density 1.40 g/cm^3^ had loose structure, the one with density 1.50 g/cm^3^ fell down immediately after stripping the mould and had a partly broken structure, and the other one with density 1.60 g/cm^3^ had a rupture at the bottom and could keep the specimen standing. However, while the unreinforced specimens with density 1.60 g/cm^3^ were moved gently, they got damaged immediately. Therefore, the unconfined compression test could not be carried out for the unreinforced specimens. These cases indicated that the unreinforced sand has insufficient unconfined compression strength to be tested in the traditional way. In this study, the approach zero values of unconfined compression strengths for all the unreinforced specimens were considered as a reference.

**Figure 6:**
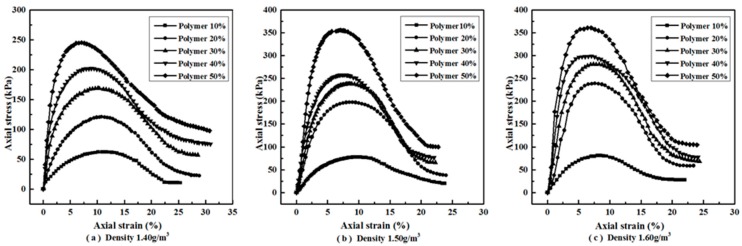
The axial stress–strain curves of reinforced specimens: (**a**) Density 1.40 g/cm^3^; (**b**) Density 1.50 g/cm^3^; (**c**) Density 1.60 g/cm^3^.

Figure 6 illustrates the axial stress–strain curves of reinforced specimens with different polymer concentrations and dry densities. It is clearly shown that all the specimens take on strain-softening ductile failure. The axial stress increased with the axial strain to a peak value, and then reduced gradually with the axial strain to a relatively stable value. The peak value and the relatively stable value are defined as peak strength and residual strength, respectively. The peak strength, residual strength, axial strain of failure (ε_f_), elasticity modulus (*E*), and failure modulus (*E*_f_) are summarized in Table 1. It can be seen from Table 1 that the peak strength of reinforced specimens in each density increased with the increasing polymer concentration. The peak strength of 245.28, 355.92, 360.97 kPa were observed at 50% polymer concentrations that were 3.93, 4.55, and 4.45 times of the ones at 10% polymer concentration with densities 1.40, 1.50, and 1.60 g/cm^3^, respectively. The peak strengths of each concentration specimens also increased with the increasing sand density—especially the specimens with higher polymer concentration. As seen in Table 1, the residual strengths of reinforced specimens in each density increased with the polymer concentration. The increment of residual strength became weakened with the increasing polymer concentration. The residual strengths of the lower concentration specimens increased with sand density, but the ones of higher concentration were almost the same at all three densities. The residual strengths of 50% polymer concentration specimens with densities 1.40, 1.50, and 1.60 g/cm^3^ were about 40%, 28%, and 29% of their peak strength, respectively. From Table 1, the axial strain of failure (ε_f_) changed with polymer concentration and density. The axial strain of failure of reinforced specimens for the same dry sand density decreased with increasing polymer concentration. It also decreased with increasing dry sand density.

**Figure 7:**
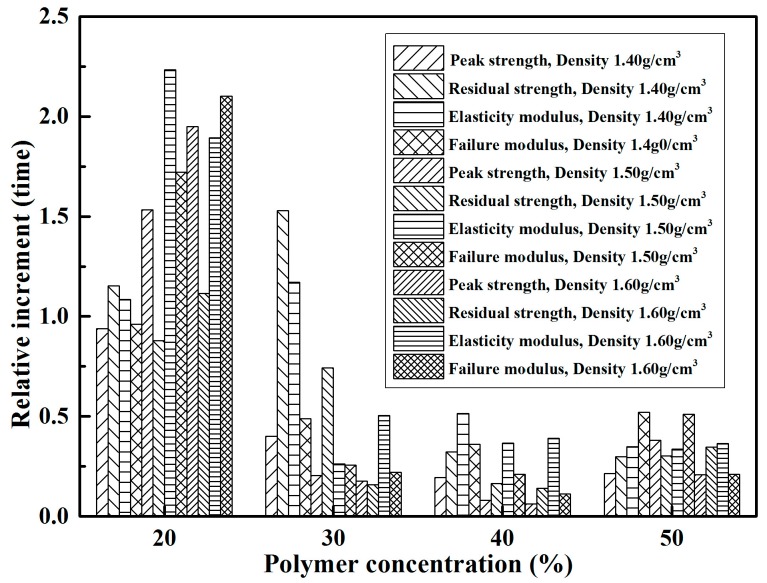
The variation of relative increment of peak strength, residual strength, elasticity modulus, and failure modulus.

**Figure 8:**
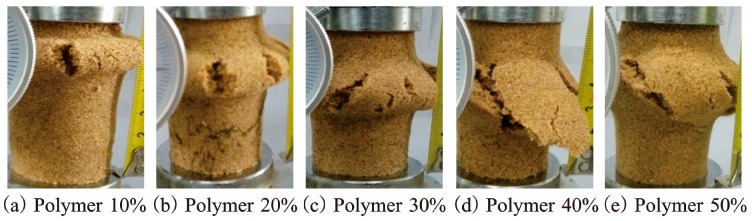
The shape of reinforced specimens after UCS test: (**a**) Polymer 10%; (**b**) Polymer 20%; (**c**) Polymer 30%; (**d**) Polymer 40%; (**e**) Polymer 50%.

Table 1 illustrates the elasticity modulus (*E*) and failure modulus (*E*_f_) of reinforced specimens in the unconfined compression test. As seen, both the elasticity modulus and failure modulus of specimens in each density increased with increasing polymer concentration, especially the elasticity modulus. The elasticity modulus of 50% polymer concentration specimens with three densities 1.40, 1.50, and 1.60 g/cm^3^ were 11.49, 11.65, and 14.94 MPa, respectively. They were 9.24, 7.45, and 8.27 times the ones at 10% polymer concentration. Additionally, the relative increment of peak strength, residual strength, elasticity modulus, and failure modulus are shown in Figure 7. As seen, the increase in these four parameters of reinforced specimens with three densities mainly occurred in the 20% polymer concentration. It should be noted that the four parameters of 10% polymer concentration specimens with three densities are having peak strengths of 62.34, 78.15, and 81.11 kPa, residual strengths of 10.46, 20.11, and 27.75 kPa, elasticity modulus of 1.24, 1.56, and 1.81 MPa, and failure modulus of 0.57, 0.81, and 1.01 MPa, respectively. After the UCS tests, the failure models of reinforced specimens are presented in Figure 8. As seen in Figure 4 and Figure 8, the failed reinforced specimens had intact form compared with the unreinforced specimens (Figure 5). The specimens reinforced with different polymer concentrations all broke at a weak cross-section that is called the “petal-model”.

### 3.2. Direct Shear Test

**Figure 9:**
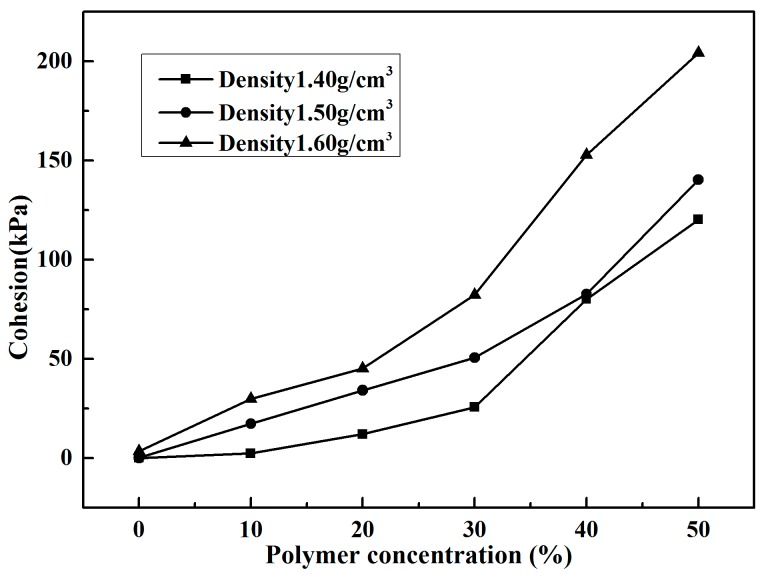
The variations of the cohesion of reinforced specimens with polymer concentration.

Direct shear tests were performed to obtain the shear strength parameters of specimens with different densities and polymer concentrations. The obtained shear strength parameters in terms of cohesion and internal friction angles are given in Table 2. It is observed that the unreinforced specimens with densities 1.40, 1.50, and 1.60 g/cm^3^ had cohesion of 0.03, 0.17, and 3.39 kPa, respectively. The polymer had a significant influence on the development of cohesion. The variation of cohesion with polymer concentrations is shown in Figure 9. As shown in Figure 9, cohesions of specimens with three densities increase with the increase in polymer concentrations. The specimens with density 1.60 g/cm^3^ and polymer concentrations of 10%, 20%, 30%, 40%, and 50% had the cohesion values of 29.81, 45.19, 82.30, 152.78, and 204.22 kPa, which are approximately 8.79, 13.33, 24.28, 45.07, and 60.24 times those of the unreinforced ones, respectively. Furthermore, the cohesions of each polymer concentration specimens increased with the increasing sand density. The specimens with polymer concentration of 10% and with densities of 1.40, 1.50, and 1.60 g/cm^3^ had cohesion values of 2.31, 17.40, and 29.81 kPa, respectively, and the ones with polymer concentration of 50% reached 120.19, 140.28, and 204.22 kPa, respectively. 

**Figure 10:**
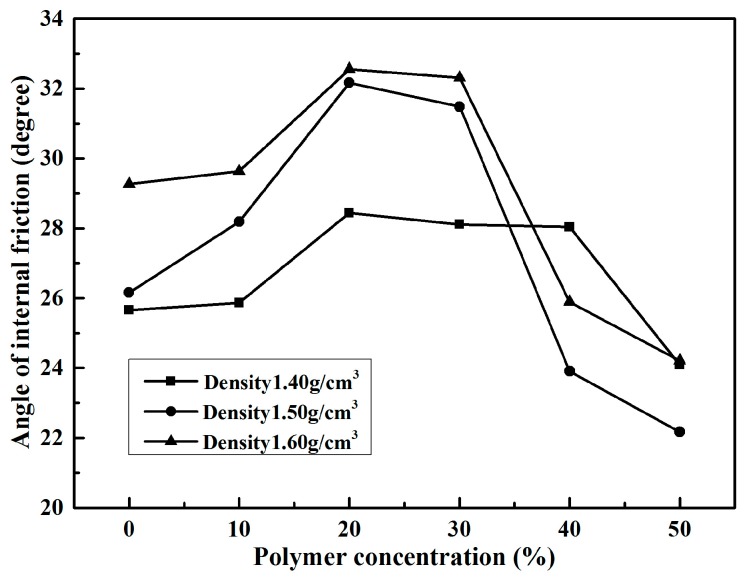
The variations of internal friction angle of reinforced specimens with polymer concentration.

**Figure 11:**
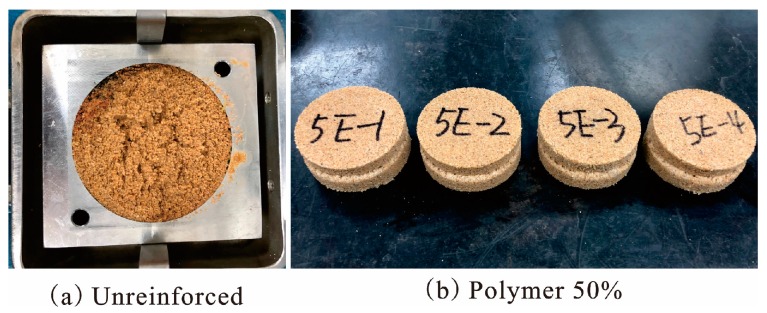
The shape of specimens after direct shear test.

It is also observed from Table 2 that the internal friction angles of specimens with these three densities had little change with increasing polymer concentration. All of the internal friction angles of unreinforced and reinforced specimens were within the range of 22° to 33°. The unreinforced specimens with densities 1.40, 1.50, and 1.60 g/cm^3^ had internal friction angles of 25.66°, 26.16°, and 29.27°, respectively. The internal friction angles of specimens with different polymer concentrations are illustrated in Figure 10. As shown in Figure 10, the internal friction angles of each density specimen increased to a peak value up to a polymer concentration of 20%, and then decreased gradually with the increasing polymer concentrations. As the polymer concentration changed from 10% to 20%, the internal friction angles of specimens with densities 1.40, 1.50, and 1.60 g/cm^3^ changed from 25.87° to 28.44°, 28.19° to 32.17°, and 29.63° to 32.56°, respectively. Subsequently, as the polymer concentration reached 50%, the internal friction angles of specimens with densities 1.40, 1.50, and 1.60 g/cm^3^ decreased to 24.09°, 22.17°, and 24.21°, respectively. After the direct shear tests, the failure models of specimens were observed (Figure 11). It is known from Figure 4 and Figure 11 that the unreinforced specimens were loose and could not get a complete direct shear test specimen. However, the reinforced specimens were intact after the tests. There was very little deformation in the failure surface of the reinforced specimens, without any apparent deformation. 

### 3.3. Tensile Test

The tensile strength mainly originates from the connection and interlocking between/among soil particles. The pull-out test could not be carried out for the unreinforced specimens due to the loose structure of sand and small cohesive force. In this study, the approach zero value of tensile strength for all the unreinforced specimens with densities 1.40, 1.50, and 1.60 g/cm^3^ was considered as a reference.

**Figure 12:**
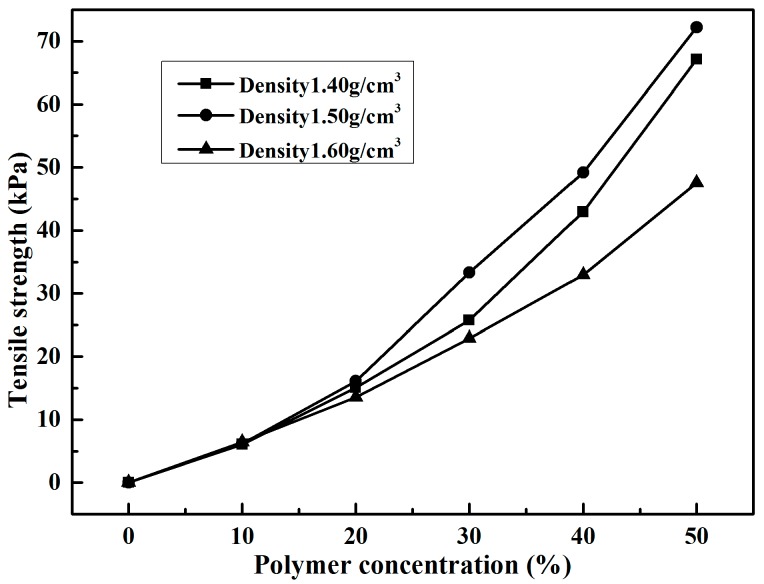
The variation of tensile strength with polymer concentration.

The pull-out test results of reinforced samples with different polymer concentrations are shown in Table 3. As seen, it is palpable that the presence of polymer had obvious effects on the tensile strength of reinforced sand. The variations of tensile strength with polymer concentration are shown in Figure 12. As shown in Figure 12, the tensile strengths of reinforced samples with three densities increased with the increasing of polymer concentration. The tensile strength of samples with density 1.40 g/cm^3^ and with polymer concentrations 10%, 20%, 30%, 40%, and 50% were 6.08, 15.08, 25.74, 42.95, and 67.11 kPa, respectively. Moreover, the samples with densities of 1.40, 1.50, and 1.60 g/cm^3^ reinforced by 10% polymer concentration had similar tensile strength values of 6.08, 6.16, and 6.42 kPa, respectively. When the additive amount of polymer concentration was higher than 10%, the tensile strengths of reinforced samples varied with sample density. The reinforced specimen with density 1.50 g/cm^3^ had the highest value of tensile strength, the one with density 1.60 g/cm^3^ had the lowest value, and the other one with density 1.40 g/cm^3^ had a middle value. The specimens with 50% polymer concentration and with densities of 1.40, 1.50, and 1.60 g/cm^3^ had tensile strengths of 67.11, 72.19, and 47.56 kPa, respectively.

**Figure 13:**
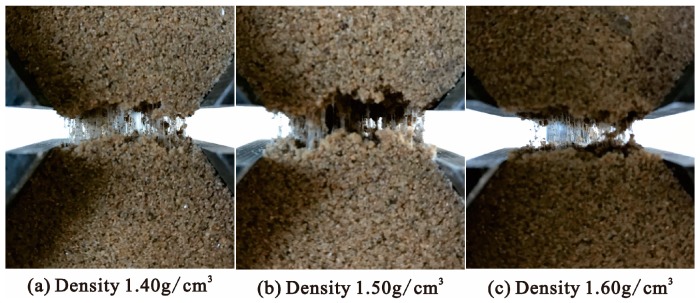
The failure module of 50% polymer-reinforced samples with different densities.

Figure 13 illustrates the failure mode of samples reinforced with 50% polymer concentration and with different densities. As shown in Figure 4 and Figure 13, after the failure of specimens, the samples with 1.40 and 1.50 g/cm^3^ densities were still tightly linked around the fracture by polymer membranes, to provide residual tensile strength. However, the samples with 1.60 g/cm^3^ density displayed a direct fracture similar to brittle failure, and had less polymer membrane.

## 4. Discussion

**Figure 14:**
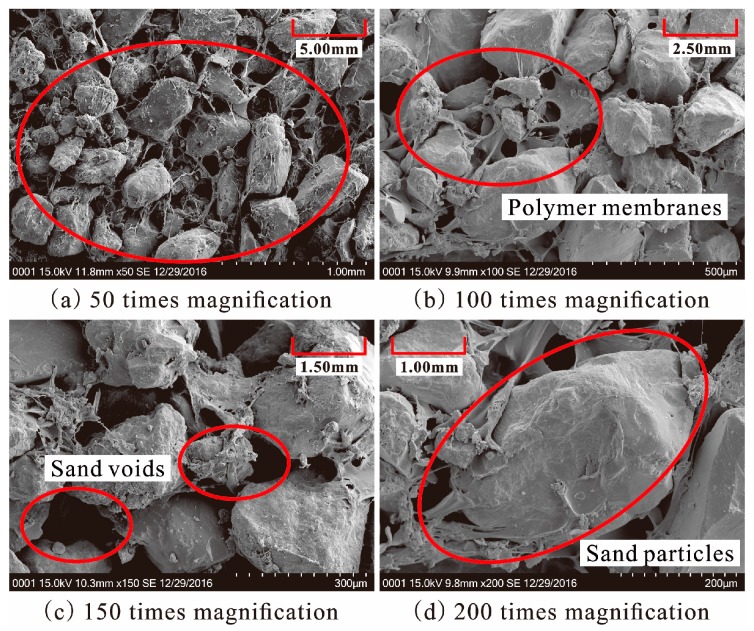
SEM images of specimen reinforced with 50% polymer: (**a**) 50 times magnification; (**b**) 100 times magnification; (**c**) 150 times magnification; (**d**) 200 times magnification.

The organic polymer used as soil stabilizer in this study reinforces sand by forming active polymer membranes between sand particles. The polymer contains a significant proportion of the long-chain macromolecule of polyurethane resin and an enormous amount of isocyanate group (–NCO). The structural formula of the polymer is given as formula (1), and its reinforcement reaction processes are

(1) $$O=C=N\;{\left[R_{1}-\mathrm{NH}-\mathrm{CO}-R_{2}-O-\mathrm{CO}-\mathrm{NH}\right]}_{n}{\;R}_{1}-N=C=O$$

(2) $$O=C=N-R-N=C=O+2H_{2}O\to \mathrm{HO}-\mathrm{CO}-\mathrm{NH}-R-\mathrm{NH}-\mathrm{CO}-\mathrm{OH}\to H_{2}N-R-{\mathrm{NH}}_{2}+2{\mathrm{CO}}_{2}.$$

(3) $$\left(n+1\right)H_{2}N-R-{\mathrm{NH}}_{2}+\mathrm{nO}=C=N-R-N=C=O\to H_{2}N\;{\left[\;R-\mathrm{NH}-\mathrm{CO}-\mathrm{NH}\;\right]}_{2n}\;R-{\mathrm{NH}}_{2}$$

given as formulas (2) and (3). When the diluted polymer solution is mixed with sand, a part of them fills up the sand voids and others are adsorbed on the surface of sand particles. This is different from cement/lime sand stabilizer, which is a new crystalline product in the form of lumps to fill most of the sand void. The active groups –NCO in formula (1) react with water in voids and on the surface of sand by chemical formulas (2) and (3) to form physicochemical bonds of polymer membrane between molecules and sand particles. With these physicochemical bonds, the polymer membranes enwrap the sand particle and interlink them to form an elastic and viscous membrane structure in the sand. The SEM images of specimen reinforced with 50% polymer are presented in Figure 14. As seen in (Figure 14a–d), the sand particles are enwrapped and connected by the polymer to form a stable structure. This structure may increase the bonding and interlocking forces between sand particles and decrease the void ratio of sand. The sand is mixed with polymer dilution to form a stable structure. The dilution concentration and specimen dry density are two important factors in the formation of a stable structure. The higher the polymer concentration, the greater the content of polymer membranes to fill sand voids and enwrap the sand particles with a resultant increase of the steadiness of stable structure in sand. So, the strength characteristics of reinforced specimens with the same dry density increase with the increasing polymer concentration (Table 1, Table 2 and Table 3, and Figure 9, Figure 10 and Figure 12).

**Figure 15:**
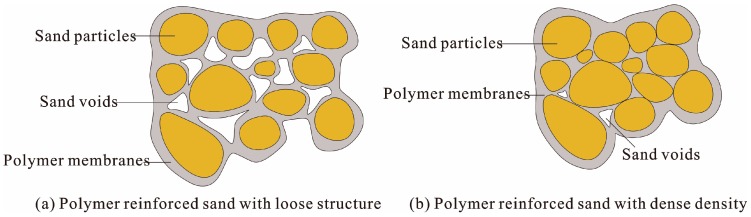
The mechanism of polymer-reinforced specimens with different dry density: (**a**) Polymer-reinforced sand with loose structure; (**b**) Polymer-reinforced with high density.

The dry density of specimen influences sand void ratio (*e*), which is a vital factor affecting the efficiency of polymer membrane in filling the sand voids and enwrapping the sand particles. Figure 15 explains the mechanism of polymer dilution reinforced specimens with different dry densities. As shown in Figure 15, the specimens with small dry density have loose structure (which helps in filling the sand voids and enwrapping the sand particles with polymer membranes), but have weak bonding and interlocking forces due to long distance between/among sand particles. The sand particles of specimens with large dry density are closely arranged, which does not help in filling the sand voids and enwrapping the sand particles. So, there are specimens with optimal density where is easy to fill the sand voids and enwrap the sand particles, and have strong bonding and interlocking forces among sand particles. It is the bonding and interlocking forces and forces between sand particles which create the pull forces when the reinforced specimens endure pull forces. However, due to small forces between sand particles, pull forces are mainly borne by bonding and interlocking forces. So, the reinforced specimens with relatively loose structure have higher tensile strength than the ones with tight structure (Figure 12). In UCS tests and direct shear tests, the specimens became denser when they were subjected to the upper pressure. So, the unconfined compression strength and shear strength of reinforced specimens with the same polymer concentration increased with the increasing density (Table 1 and Table 2, and Figure 9). 

## 5. Conclusions

In order to evaluate the efficacy of polyurethane soil stabilizer on the strength characteristics of reinforced sand, laboratory trials of unconfined compression test, direct shear test, and tensile test were performed. The test results and reinforcement mechanism were analysed. Based on the results of the tests presented herein, the main conclusions can be summarized as follows:

1. The addition of polymer had significant effects on the strength characteristics of reinforced specimens. The unconfined compressive strength, cohesion, and tensile strength of specimens with the same dry density increased with the increasing polymer concentration. Moreover, specimen dry density is an important factor for the reinforced specimen strength characteristics. The unconfined compressive strength and shear strength of specimens reinforced with same polymer concentration increased with increasing sand density, and the reinforced specimen with density 1.50 g/cm^3^ had the highest value of tensile strength, the one with density 1.60 g/cm^3^ had the lowest value, and the other one with density 1.40 g/cm^3^ had a moderate value.
2. When the organic polymer solution was applied to sand, the polymer membranes formed by the mixture of polymer and water enwrapped the sand particles and interlinked them to form a stable structure. The higher the polymer concentration, the greater the content of polymer membranes to fill the sand voids and enwrap the sand particles to keep the sand structure stable. The efficiency of this phenomenon is influenced by dry density, and at an optimal density of sand, the process of filling the sand voids and enwrapping the sand particles gets easier, and strong bonding and interlocking forces among sand particles are observed. These results provide a reasonable and effective theoretical basis for the reinforcement of the sand soil area.

## Figures and Tables

**Table 1:** Results of unconfined compression strength tests.

Serial Number	Polymer Concentration (%)	Dry Density (g/cm^3^)	Void Ratio *e_s_*	Relative Density *D*_r_ (%)	Peak Strength (kPa)/Standard Deviations (kPa)	Residual Strength (kPa)/Standard Deviations (kPa)	Axial Strain of Failure (%)/Standard Deviations (kPa)	Elasticity Modulus *E* (MPa)/Standard Deviations (kPa)	Failure Modulus *E*_f_ (MPa)/Standard Deviations (kPa)
S1	water	1.40	0.89	21.05	/	/	/	/	/
S2	10	1.40	0.89	21.05	62.34/2.37	10.46/1.47	10.88/0.05	1.244/0.05	0.573/0.00
S3	20	1.40	0.89	21.05	120.83/2.35	22.52/1.34	10.75/0.17	2.594/0.10	1.124/0.01
S4	30	1.40	0.89	21.05	169.22/3.82	56.95/1.61	10.11/0.29	5.629/0.29	1.674/0.02
S5	40	1.40	0.89	21.05	201.94/3.20	75.29/1.61	8.87/0.38	8.526/0.34	2.277/0.05
S6	50	1.40	0.89	21.05	245.28/3.44	97.73/2.21	7.08/0.40	11.494/0.78	3.464/0.07
S7	water	1.50	0.76	55.26	/	/	/	/	/
S8	10	1.50	0.76	55.26	78.15/3.28	20.11/1.34	9.68/0.10	1.564/0.05	0.807/0.00
S9	20	1.50	0.76	55.26	197.96/3.63	37.77/2.89	9.01/0.05	5.057/0.13	2.197/0.04
S10	30	1.50	0.76	55.26	238.65/3.22	65.87/1.72	8.65/0.16	6.380/0.15	2.759/0.05
S11	40	1.50	0.76	55.26	257.92/4.92	76.63/2.69	7.72/0.23	8.722/0.25	3.341/0.08
S12	50	1.50	0.76	55.26	355.92/3.44	99.77/2.31	7.05/0.20	11.654/0.67	5.049/0.17
S13	water	1.60	0.65	84.21	/	/	/	/	/
S14	10	1.60	0.65	84.21	81.11/4.28	27.75/1.83	8.02/0.22	1.806/0.04	1.011/0.04
S15	20	1.60	0.65	84.21	239.25/3.22	58.67/2.65	7.63/0.14	5.227/0.18	3.136/0.06
S16	30	1.60	0.65	84.21	281.69/3.43	67.96/1.33	7.36/0.07	7.868/0.26	3.827/0.07
S17	40	1.60	0.65	84.21	298.92/4.99	77.51/2.70	7.03/0.17	10.945/0.46	4.252/0.13
S18	50	1.60	0.65	84.21	360.97/3.15	104.27/1.79	7.01/0.11	14.940/0.56	5.149/0.19

**Table 2:** Results of direct shear tests.

Serial Number	Polymer Concentration (%)	Dry Density (g/cm^3^)	Void Ratio *e_s_*	Relative Density *D*_r_ (%)	Cohesion (kPa)/Standard Deviations (kPa)	Angle of Internal Friction (degree)/Standard Deviations (kPa)
S19	water	1.40	0.89	21.05	0.03/0.00	25.66/0.35
S20	10	1.40	0.89	21.05	2.31/0.13	25.87/0.98
S21	20	1.40	0.89	21.05	12.12/0.35	28.44/0.31
S22	30	1.40	0.89	21.05	25.67/0.94	28.11/0.62
S23	40	1.40	0.89	21.05	80.09/0.50	28.04/0.41
S24	50	1.40	0.89	21.05	120.19/2.12	24.09/0.73
S25	water	1.50	0.76	55.26	0.17/0.00	26.16/0.30
S26	10	1.50	0.76	55.26	17.40/0.46	28.19/0.03
S27	20	1.50	0.76	55.26	34.16/0.78	32.17/0.89
S28	30	1.50	0.76	55.26	50.58/1.21	31.48/0.42
S29	40	1.50	0.76	55.26	82.59/2.66	23.91/0.02
S30	50	1.50	0.76	55.26	140.28/2.79	22.17/0.62
S31	water	1.60	0.65	84.21	3.39/0.04	29.27/0.26
S32	10	1.60	0.65	84.21	29.81/1.23	29.63/0.60
S33	20	1.60	0.65	84.21	45.19/1.11	32.56/0.67
S34	30	1.60	0.65	84.21	82.30/1.79	32.31/0.57
S35	40	1.60	0.65	84.21	152.78/2.34	25.89/0.99
S36	50	1.60	0.65	84.21	204.22/3.15	24.21/0.29

**Table 3:** Results of tensile tests.

Serial Number	Polymer Concentration (%)	Dry Density (g/cm^3^)	Void Ratio *e_s_*	Relative Density *D*_r_ (%)	Tensile Strength (kPa)/Standard Deviations (kPa)
S37	water	1.40	0.89	21.05	0.00/0.00
S38	10	1.40	0.89	21.05	6.08/0.13
S39	20	1.40	0.89	21.05	15.08/0.24
S40	30	1.40	0.89	21.05	25.74/0.58
S41	40	1.40	0.89	21.05	42.95/1.27
S42	50	1.40	0.89	21.05	67.11/1.89
S43	water	1.50	0.76	55.26	0.00/0.00
S44	10	1.50	0.76	55.26	6.16/0.14
S45	20	1.50	0.76	55.26	16.11/0.56
S46	30	1.50	0.76	55.26	33.33/0.66
S47	40	1.50	0.76	55.26	49.19/0.57
S48	50	1.50	0.76	55.26	72.19/1.45
S49	water	1.60	0.65	84.21	0.00/0.00
S50	10	1.60	0.65	84.21	6.42/0.12
S51	20	1.60	0.65	84.21	13.57/0.25
S52	30	1.60	0.65	84.21	22.92/0.45
S53	40	1.60	0.65	84.21	32.93/0.57
S54	50	1.60	0.65	84.21	47.56/1.29
